# Sesquiterpene Lactones and Flavonoids from *Psephellus pyrrhoblepharus* with Antiproliferative Activity on Human Gynecological Cancer Cell Lines

**DOI:** 10.3390/molecules24173165

**Published:** 2019-08-30

**Authors:** Pelin Tastan, Zsuzsanna Hajdú, Norbert Kúsz, István Zupkó, Izabella Sinka, Bijen Kivcak, Judit Hohmann

**Affiliations:** 1 Department of Pharmacognosy, Faculty of Pharmacy, Ege University, 35040 Bornova/İzmir, Turkey; 2 Institute of Pharmacognosy, Interdisciplinary Excellence Centre, University of Szeged, 6720 Szeged, Hungary; 3 Institute of Pharmacodynamics and Biopharmacy, Interdisciplinary Excellence Centre, University of Szeged, 6720 Szeged, Hungary; 4 Interdisciplinary Centre of Natural Products, University of Szeged, 6720 Szeged, Hungary

**Keywords:** *Psephellus pyrrhoblepharus*, Asteraceae, sesquiterpene lactones, chlorojanerin, 19-deoxychlorojanerin, gynecological cancer cells, antiproliferative activity

## Abstract

Multistep chromatographic separations of the chloroform extract of the Turkish endemic plant *Psephellus pyrrhoblepharus* (Boiss.) Wagenitz (syn. *Centaurea pyrrhoblephara* Boiss.) resulted in the isolation of six guaianolid-type sesquiterpenes, chlorojanerin (**1**), 19-deoxychlorojanerin (**2**), 15-hydroxyjanerin (**3**), aguerin B (**4**), cynaropicrin (**5**), eleganin (**6**); three flavonoids, apigenin, 6-methoxyluteolin and jaceosidine; two glycosides, benzyl-1-*O-*β-d-glucoside and 3(*Z*)-hexenyl-1-*O-*β-d-glucoside; and the coumarin scopoletin. The structures were established by the interpretation of their ESI-MS and 1D and 2D NMR data including ^1^H-NMR, JMOD, ^1^H,^1^H-COSY, HSQC, HMBC, and NOESY experiments. All compounds were isolated for the first time from *P. pyrrhoblepharus*. Compounds **1**–**6**, the isolated flavonoids and scopoletin were evaluated for their antiproliferative activities on human gynecological cancer cell lines (SiHa, HeLa, and MDA-MB-231 cells) using the MTT (3-(4,5-dimethylthiazol-2-yl)-2,5-diphenyltetrazolium bromide) assay. Chlorojanerin (**1**), 19-deoxychlorojanerin (**2**), aguerin B (**4**), cynaropicrin (**5**), eleganin (**6**) were shown to have noteworthy effects on all of the tested cell lines, while apigenin, jaceosidine, and 6-methoxyluteolin were moderately active on HeLa cells. The highest activities were demonstrated by the chlorine-containing derivatives chlorojanerin (**1**) and 19-deoxychlorojanerin (**2**) with IC_50_ values of 2.21 and 2.88 µM, respectively, against the triple negative breast cancer model MDA-MB-231 cells.

## 1. Introduction

The genus *Psephellus* Cass., belonging to the Asteraceae family, tribe Cynareae, includes approximately 80 species. In Turkey, this genus is represented by 32 species. Some of these are new members of the genus as some sections of the genus *Centaurea* have been transferred into the genus *Psephellus* in recent years*. Psephellus pyrrhoblepharus* (Boiss.) Wagenitz (syn. *Centaurea pyrrhoblephara* Boiss.) is an endemic plant of Turkey, and grows in the Middle and East Black Sea Region, Upper Red River Area, and Upper Fırat Area of the country [1,2,3]. *Psephellus* species such as *P. appendicigerus, P. erzincani*, and *P. karduchorum* have been used in traditional medicine as wound healing agents and in the treatment of abscesses [4,5,6,7]. Basal leaves of *P. karduchorum* have been used as foodstuff and eaten in fresh form [6]. 

A systematic survey of plants used against cancer by J. Hartwell reported that some species of the tribe Cynareae (Carduu*s acanthoides*, *Cynara scolymus*, *Cirsium acarna*. C. ferox, *Arctium lappa*, A. minus, *Centaurea acaulis, C. calcitrapa*, *C. centaurium*, *C. cyanus*, *C. monantha*, *C. nigra*, *C. salonitana*, and *C. scabiosa*) were applied against cold tumors, carcinomas, tumors of the eye and throat, uterine fibroids, and induration of spleen and liver in the form of decoctions, plasters, liniments, and pastes [8]. Ethnopharmacological studies have revealed that *Centaurea ornata* and *C. repens* have been used in the treatment of cancer-related diseases [9,10]. A wide range of pharmacological studies have been carried out on *Centaurea*, *Arctium*, *Cirsium*, and *Psephellus* species to examine their antitumor activities, and significant cell proliferation inhibitory properties have been demonstrated in many cases [11,12,13]. Bioactivity-guided phytochemical investigations on the active extracts revealed that, in general, the presence of sesquiterpene lactones, flavonoids, and lignans as compounds responsible for the antiproliferative effects [14,15,16]. 

The genus *Psephellus* was previously reported to be a rich source of sesquiterpenes and lignans [17,18]; moreover *P. pyrrhoblepharus* was published to accumulate essential oil, containing monoterpene and sesquiterpene hydrocarbons, and carbonylic compounds in substantial quantities [19].

**Figure 1:**
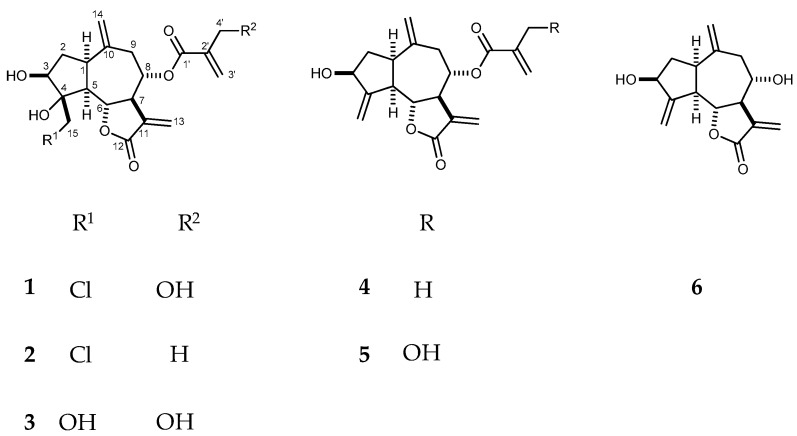
Structure of the isolated sesquiterpene lactones **1**–**6**.

The present study aimed at the phytochemical profiling of the Turkish endemic plant *P. pyrrhoblepharus*, with special focus on the non-volatile compounds with antitumor potency. Multistep chromatographic separation of the chloroform extract of the aerial plant parts was carried out, resulting in the isolation of six guaianolid-type sesquiterpenes, chlorojanerin (**1**), 19-deoxychlorojanerin (**2**), 15-hydroxyjanerin (**3**), aguerin B (**4**), cynaropicrin (**5**), eleganin (**6**) (Figure 1); three flavonoids, apigenin, 6-methoxyluteolin and jaceosidine; two glycosides, benzyl-1-*O-*β-d-glucoside and 3(*Z*)-hexenyl-1-*O-*β-d-glucoside; and the coumarin scopoletin. Compounds **1**–**6**, the isolated flavonoids and scopoletin were evaluated for their antiproliferative activities using the MTT assay. Human gynecological malignant cell lines isolated from cervical (SiHa, HeLa) and female breast cancers (MDA-MB-231) were utilized with regard to previous investigations where they were found to be sensitive against metabolites of Cynareae plants [11,20,21,22,23]. In the present assay, chlorojanerin (**1**), 19-deoxychlorojanerin (**2**), aguerin B (**4**), cynaropicrin (**5**), and eleganin (**6**) were shown to have noteworthy effects on all of the tested cell lines, while apigenin, jaceosidine, and 6-methoxyluteolin were moderately active on HeLa cells.

## 2. Results and Discussion

Antiproliferative testing of species belonging to tribe Cynareae [11] and previous screening results on *P. pyrrhoblepharus* [12] initiated the present work, aiming at the identification of compounds of *P. pyrrhoblepharus* with tumor cell growth inhibitory effect. The aerial parts of the plant were extracted with MeOH at room temperature and, after concentration, the extract was partitioned between *n-*hexane, CHCl_3_, and H_2_O. The CHCl_3_ phase was subjected to a multistep chromatographic separation and purification procedure including CC, VLC, RPC, gel filtration, and preparative TLC, resulting in the isolation of pure compounds **1**–**6** (Figure 1), apigenin, 6-methoxyluteolin, jaceosidine, benzyl-1-*O-*β-d-glucoside, 3(*Z*)-hexenyl-1-*O-*β-d-glucoside, and scopoletin. The structures of the pure compounds were established by means of NMR and mass spectroscopy including ^1^H-NMR (Figures S1–S10), JMOD, ^1^H,^1^H-COSY, HSQC, HMBC, and NOESY experiments and ESI-MS. For all compounds, complete and unambiguous assignments of the NMR chemical shifts of protons and carbons were achieved. The previously unpublished ^1^H- and ^13^C-NMR data of compounds **1**–**6** and benzyl-1-*O*-β-d-glucoside in CD_3_OD, and jaceosidine in DMSO-*d_6_* are listed in Table 1 and Table 2, and in Section 3. All compounds were isolated for the first time from *P. pyrrhoblepharus*.

Chlorojanerin (**1**), 19-deoxychlorojanerin (**2**), 15-hydroxyjanerin (**3**), aguerin B (**4**), cynaropicrin (**5**), eleganin (**6**), apigenin, 6-methoxyluteolin, jaceosidine, and scopoletin were investigated for their antiproliferative activities on SiHa, HeLa, and MDA-MB-231 cells using the MTT assay.

Based on the determined antiproliferative activities (Table 3) it can be concluded that, with the exception of compound **3**, the isolated sesquiterpenes have comparable efficacy to or are even more effective than the reference agent cisplatin against all of the cell tested cell lines. The highest activities were found against the triple negative breast cancer model MDA-MB-231 (IC_50_ 2.21–6.86 µM); the chlorine-containing derivatives chlorojanerin (**1**) (IC_50_ 2.21 µM) and 19-deoxychlorojanerin (**2**) (IC_50_ 2.88 µM) were more effective than the well-known sesquiterpene lactone cynaropicrin (**5**) (IC_50_ 4.80 µM) [20]. The very close IC_50_ values of compound pairs **1** and **2**, and **4** and **5** indicated that the side chain methacrylate or 4-hydroxymethacrylate in position C-8 have no major influence on the activity.

Interestingly, 15-hydroxyjanerin (**3**), a close analogue of chlorojanerin (**1**), did not elicit any considerable activity, indicating that 15-hydroxy group significantly decreases the antiproliferative potency. The same observation was published by Iranshahy et al. [21], who reported 15-hydroxyjanerin (**3**) (=aitchisonolide) to be ineffective on a set of human tumor cell lines, MCF-7, MCF-7/MX, PC-3, HL-60, Jurkat, and HEK. The non-esterified sesquiterpene eleganin (**6**) exerted reasonable action against the SiHa and MDA-MB-231 cells. Concerning the isolated flavonoids, jaceosidine exhibited a modest action on all cell lines (i.e., 40–75% cell growth inhibition at 30 μM), the effects of apigenin and 6-methoxyluteolin were similar but restricted to HeLa cells, while the only tested coumarin, scopoletin, was ineffective on all cell lines. 

In conclusion, this study demonstrates that *P. pyrroblepharus* is a rich source of sesquiterpene lactones and flavonoids with antiproliferative activities. The chlorinated sesquiterpenes are particularly considered promising and suitable for additional experiments in order to describe the potential mechanism of the detected action. Sesquiterpene lactones represent a main focus of anticancer research; their clinical relevance can be demonstrated by compounds newly introduced in clinical practice such as arglabin, the dimethylamino adduct of which is a registered antitumor drug in the Republic of Kazakhstan [24], and by compounds presently under clinical assessment (artemisinin, parthenolide, and thapsigargin) [25]. Sesquiterpenes are known to exhibit considerable antitumor activity; it is generally believed that the α-methylene-γ-lactone moiety is the functional group responsible for the biological activity due to its interaction with biological nucleophiles. Their ability to inhibit DNA and protein synthesis has been proven for many compounds. Cynaropicrin (**5**) was reported to increase the relative expression levels of the important G2/mitosis checkpoint proteins, p21^Waf1/Cip1^, p-Tyr15-CDK1, and cyclin B1, which may be related to the G2 cell cycle arrest in MDA-MB-231 breast cancer cells. This compound downregulated the p-Ser473-Akt protein level without affecting the total Akt1 protein level. Cynaropicrin did not affect caspase-3 activity [20]. 

Tumor cell selectivity was also investigated, and in the case of chlorojanerin (**1**), cynaropicrin (**5**), and janerin, it was found that they are toxic to VERO cells (IC_50_ 5.9–6.7 µg/mL) [26]. Cynaropicrin was reported to reduce the 48 h-cell viability of human skin fibroblast Detroit 551 cells (IC_50_ 9.11 µg/mL) [27]. 15-Hydroxyjanerin was revealed to be inactive (IC_50_ > 50 µg/mL) against the HEK cell line [21].

In an anti-angiogenic study of sesquiterpene lactones, cynaropicrin (**5**), aguerin B (**4**), artemisinin, thapsigargin, and parthenolide have demonstrated the ability to inhibit angiogenesis in vitro and in ovo by inhibiting HUVEC proliferation, microvessel formation, and proliferation of human artery endothelial cells. In addition, cynaropycrin and aguerin B exhibited remarkable angio-inhibitory effects in the CAM assay [23].

## 3. Materials and Methods 

### 3.1. General Procedures

NMR spectra were recorded in CDCl_3_ on a Bruker Avance DRX 500 spectrometer (Billerica, MA, USA) at 500 MHz (^1^H) and 125 MHz (^13^C). In the ^1^H,^1^H-COSY, HSQC, and HMBC experiments, gradient-enhanced versions were employed. Data were recorded and processed with the MestReNova *v*6.0.2-5475 software (Santiago de Compostela, Spain). Chemical shifts are expressed in parts per million, and coupling constant (*J*) values are reported in Hz. Mass spectra were recorded on a Thermo Q Exactive mass spectrometer (Waltham, MA, USA) equipped with an ESI electrospray source. Data were recorded and processed with the Thermo Xcalibur software (version 4.1, Waltham, MA, USA). 

For column chromatography, silica gel 60 (0.063–0.200 mm) (70–230 mesh ASTM, Merck, Darmstadt, Germany) was applied. For vacuum-liquid chromatography (VLC), silica gel (60 GF₂₅₄, 15 µm, Merck) was used, and for gelfiltration, we used Sephadex LH-20 (GE Healthcare, Uppsala, Sweden). Pre-coated normal phase silica gel plates (60 F_254_, 0.25 mm, Merck) and reversed phase silica gel plates (60 RP-18 F₂₅₄s, Merck) were used for thin-layer chromatographic (TLC) analyses and preparative TLC. Spots were visualized by heating (105 °C) after spraying the plates with concentrated H_2_SO_4_. Rotational planar chromatography (RPC) was performed on self-coated silica gel (60 GF₂₅₄, 15 µm, Merck) plates using a Chromatotron instrument (Harrison Research, T-Squared Technology, Inc., San Bruno, CA, USA).

### 3.2. Plant Material

*Psephellus pyrrhoblepharus* (Boiss.) Wagenitz was collected during the flowering period from rocky areas of Buzluk Cave, Harput, Elazığ, Turkey in 2012, at 1500 m. The plant was identified by Assoc. Prof. Ugur Cakilcioglu from Munzur University, Tunceli. A voucher specimen (No. 1464) was deposited in the Herbarium of Department of Pharmacognosy, Faculty of Pharmacy, Ege University, Izmir, Turkey.

### 3.3. Extraction and Isolation

The aerial parts of the plant were dried (750 g) and percolated with 20 L methanol. After evaporation, the MeOH extract was suspended in 1 L MeOH:H_2_O (1:1) and extracted with *n*-hexane (5 × 0.5 L) and CHCl_3_ (5 × 0.5 L). The CHCl_3_ extract was evaporated under reduced pressure to dryness, yielding 19.1 g of oily material. This extract was chromatographed over a silica gel column (800 g) using *n*-hexane:acetone:MeOH mixtures (9:1:0, 8:2:0, 7:3:0, 6:4:0, 5:5:0, 5:5:0.3, 1:1:0.1, 5:5:1, 5:5:2, and 1:1:1, 800 mL each, volume of the fractions was 50 mL). Fractions collected were combined to fractions A–D after TLC monitoring on silica gel 60 F_254_ using CHCl_3_:MeOH:acetone (9:2:1, 96:4:0), *n*-hexane:EtOAc:EtOH (30:30:1), and toluene:EtOAc:HCOOH (5:4:1) as developing systems. Fraction A (60 mg) was subjected to preparative silica gel TLC separation with toluene:EtOAc:MeOH 12:7:1 as the developing system. Compound at Rf 0.35 was further purified on Sephadex LH-20 gel with a mixture of acetone:MeOH 1:1, affording compound **4** (8.9 mg). Fraction B (162 mg) was subjected to preparative TLC using the solvent system toluene:EtOAc:MeOH 12:7:1 to obtain compound **2** (Rf = 0.27, 12 mg). Fraction C (3.9 g) was separated by vacuum-liquid chromatography (VLC) on silica gel with increasing polarity of CHCl_3_:MeOH (10:0, 99:1, 98:2, 95:5, and 90:10, 50 mL each). Ten fractions (C_1–10_) were obtained after TLC monitoring in this separation, all of them were subjected to Sephadex LH-20 gel filtration using acetone:MeOH 1:1 as the eluent, yielding apigenin (14.5 mg) from fraction C_1,2_, and after preparative TLC purification on silica gel using toluene:EtOAc:MeOH (12:7:1) as the developing system, jaceosidine (6.7 mg) and scopoletin (4.1 mg) from fraction C_3–5_. In addition, compounds **1** (6.4 mg), 5 (25.3 mg) and 6 (13.6 mg) were isolated from fractions C_3–7_ in pure form by using rotational planar chromatography (RPC) with toluene:EtOAc:MeOH 120:75:5 (100 mL), and 60:35:5 (100 mL) as the developing solvent systems. Fraction D (2 g) was subjected to VLC with the gradient system of *n*-hexane:EtOAc:MeOH (6:3:0.5, 6:3:1, 6:3:1.5, and 6:3:4, 200 mL each), resulting in subfractions I and II. Subfraction I (0.7 g) was separated by RPC on silica gel using CH_2_Cl_2_:MeOH (10:0 and 9:1, 80 mL each), affording 6-methoxyluteolin (3.7 mg). Subfraction II (1.1 g) was subjected to Sephadex LH-20 gel filtration using acetone:MeOH 1:1 and then to RPC on silica gel using CH_2_Cl_2_:MeOH (10:0, 98:2, 96:4, 94:6, 92:8, 90:10, and 80:20, 100 mL each), resulting in compound **3** (18.3 mg) and a mixture of two compounds. This mixture could be separated by preparative RP-TLC with methanol:water 7:3, and HPLC with *n*-hexane:isopropanol 8:2 obtaining benzyl-1-*O*-β-d-glucoside (t*_R_* = 12.1 min, 3 mg) and 3(*Z*)-hexenoyl-1-*O*-β-d-glucoside (t*_R_* = 12.6 min, 6.7 mg).

*Chlorojanerin* (**1**): colorless amorphous solid; ESI-MS (positive): *m/z* 416 [M + H + NH_3_]^+^, 399 [M + H]^+^, 297 [M + H − C_4_H_6_O_3_]^+^, 279 [M + H − C_4_H_6_O_3_ − H_2_O]^+^; ^1^H-NMR (MeOH-*d_4_*, 500 MHz) see Table 1; ^13^C-NMR (MeOH-*d_4_*, 125 MHz) see Table 2.

*19-Deoxychlorojanerin* (**2**): colorless amorphous solid; ^1^H-NMR (MeOH-*d_4_*, 500 MHz) see Table 1; ^13^C-NMR (MeOH-*d_4_*, 125 MHz) see Table 2.

*15-Hydroxyjanerin* (**3**): colorless amorphous solid; ESI-MS (positive): *m/z* 398 [M + H + NH_3_]^+^, 381 [M + H]^+^, 279 [M + H − C_4_H_6_O_3_]^+^, 261 [M + H − C_4_H_6_O_3_ − H_2_O]^+^, 243 [M + H − C_4_H_6_O_3_ − 2 × H_2_O]^+^; ^1^H-NMR (MeOH-*d_4_*, 500 MHz) see Table 1; ^13^C-NMR (MeOH-*d_4_*, 125 MHz) see Table 2.

*Aguerin B* (**4**): colorless oil; ^1^H-NMR (MeOH-*d_4_*, 500 MHz) see Table 1.

*Cynaropicrin* (**5**): colorless oil; ^1^H-NMR (MeOH-*d_4_*, 500 MHz) see Table 1; ^13^C-NMR (MeOH-*d_4_*, 125 MHz) see Table 2.

*Eleganin* (**6**): colorless oil; ^1^H-NMR (MeOH-*d_4_*, 500 MHz) see Table 1; ^13^C-NMR of 1–3, 5 and 6 (MeOH-*d_4_*, 125 MHz) see Table 2.

*Jaceosidine*: yellow powder; ^1^H-NMR (DMSO-*d_6_*, 500 MHz, δ ppm) 6.88 (1H, s, H-3), 6.60 (1H, s, H-8), 7.55 (2H, m, H-2′, H-6′), 6.93 (1H, d, *J* = 8.5 Hz, H-5′), 3.75 (3H, s, 6-OCH_3_), 3.89 (3H, s, 3′-OCH_3_); ^13^C-NMR (DMSO-*d_6_*, 125 MHz, δ ppm) 163.6 (C-2), 102.7 (C-3), 182.1 (C-4), 152.7 (C-5), 131.4 (C-6), 157.7 and 152.5 (C-7, C-9), 94.4 (C-8), 103.9 (C-10), 121.6 (C-1′), 110.2 (C-2′), 148.0 (C-3′), 150.7 (C-4′), 115.8 (C-5′), 120.3 (C-6′), 56.0 (3′-OCH_3_).

*6-Methoxyluteolin*: yellow powder; ^1^H-NMR and ^13^C-NMR (MeOH-*d_4_*, 500/125 MHz) data are identical with the published data [28].

*Apigenin* and *scopoletin* were identified on the basis of the ^1^H-NMR spectra and by using authentic materials.

*Benzyl-1-O-β-d-glucoside*: amorphous solid; ^1^H-NMR (MeOH-*d_4_*, 500 MHz, δ ppm) 7.42 (2H, d, *J* = 7.3 Hz, H-2, H-6), 7.32 (2H, t, *J* = 7.3 Hz, H-3, H-5), 7.24 (1H, t, *J* = 7.2 Hz, H-4), 4.93 (1H, d, *J* = 11.8 Hz, H-7a), 4.67 (1H, d, *J* = 11.8 Hz, H-7b), 4.35 (1H, d, *J* = 7.8 Hz, H-1′), 3.33 (1H, t, *J* = 8.2 Hz, H-3′), 3.29 and 3.25 (1H and 2H, each m, H-2′, H-4′, H-5′), 3.89 (1H, d, *J* = 12.2 Hz, H-6′a), 3.69 (1H, dd, *J* = 12.0 and 5.4 Hz, H-6′b); ^13^C-NMR (MeOH-*d_4_*, 125 MHz, δ ppm) 139.1 (C-1), 129.3 (C-2, C-6), 129.2 (C-3, C-5), 128.7 (C-4), 71.8 (C-7), 103.3 (C-1′), 75.2 (C-2′), 78.1 (C-3′), 71.7 (C-4′), 78.0 (C-5′), 62.8 (C-6′).

*3-(*Z*)-Hexenyl-1-O-β-d-glucoside*: amorphous solid; ^1^H-NMR and ^13^C-NMR (MeOH-*d_4_*, 500/125 MHz) identical with the published data [29].

### 3.4. Antiproliferative Assay

The antiproliferative effects of the isolated natural products were determined by means of the MTT ([3-(4,5-dimethylthiazol-2-yl)-2,5-diphenyltetrazolium bromide]) assay on a panel of human adherent cancer cells of gynecological origin. HeLa (HPV 18+ cervix carcinoma) and MDA-MB-231 (triple-negative breast cancer) cell lines were purchased from the European Collection of Cell Cultures (Salisbury, UK) while SiHa (HPV 16+ cervix carcinoma) was obtained from the American Tissue Culture Collection (Manassas, VA, USA). The cells were maintained in minimum essential medium (MEM) supplemented with 10% fetal calf serum (FCS), 1% non-essential amino acids, and 1% penicillin-streptomycin. All media and supplements for these experiments were obtained from Lonza Group Ltd. (Basel, Switzerland). The cells were maintained at 37 °C in a humidified atmosphere containing 5% CO_2_. The assays were performed as published previously [30]. Briefly, cells were seeded onto 96-well plates at a density of 5000 cells/well and allowed to stand overnight, after which the medium containing the tested compounds at 10 and 30 μM was added. After a 72-h incubation, viability of the treated cells was determined by the addition of 20 µL of MTT solution (5 mg/mL). The precipitated formazan crystals were solubilized in dimethylsulfoxide and the absorbance was determined at 545 nm with an ELISA reader. In the case of the most effective compounds (i.e., exhibiting at least 25% of cancer cell growth inhibition at 10 μM), the assays were repeated with a set of dilutions (0.3–30 µg/mL) in order to determine the IC_50_ values by means of the computer program GraphPad Prism 4.03 (San Diego, CA, USA). Two independent experiments were performed with five wells for each condition. Cisplatin (Ebewe GmbH, Unterach, Austria), a clinically used anticancer agent, was used as a reference compound.

## 4. Conclusions

Ethnopharmacological relevance of *Psephellus* species and accumulation of antitumor compounds in genera *Centaurea*, *Arctium, Cirsium*, and *Psephellus* belonging to tribe Cynareae initiated the present phytochemical investigation that resulted in the isolation and structural elucidation of 12 secondary metabolites. The isolated compounds were subjected to antiproliferative screening using a set of human gynecological cancer cell lines, and several sesquiterpenes exhibited significant antiproliferative activities against SiHa, HeLa, and MDA-MB-231 cells with IC_50_ values ranging from 2.21 to 19.62 μM. 19-Deoxychlorojanerin was first investigated in our experiment for antiproliferative activity. Chlorojanerin, hydroxyjanerin, and eleganine were previously tested only against other tumor cell lines, melanoma (SK-MEL, LOX-IMVI), epidermoid (KB), ductal (BT-549) and ovarian carcinomas (SK-OV-3), non-small lung cancer (A549), breast (MCF-7), prostate (PC-3), and colorectal (HCT-15) adenocarcinomas [22,26,31]. Hydroxyjanerin was investigated additionally on HeLa cells [32]. Cynaropicrin and aguerin B are well-investigated sesquiterpenes, whose antitumor effects have been studied in detail including their mechanism of action [20,22,23,31]. The most promising compounds isolated in the present experiment from *P. pyrrhoblepharus* are the chlorinated sesquiterpenes chlorojanerin (**1**) (IC_50_ from 2.21 to 11.37 μM) and 19-deoxychlorojanerin (**2**) (IC_50_ from 2.88 to 5.54 μM) with higher activities than that of cynaropicrin; these compounds are attractive substrates for further studies to explore their mechanism of action, selectivity, and safety profile.

## Figures and Tables

**Table 1:** ^1^H-NMR data of compounds **1**–**6** [MeOH-*d_4_*, 500 MHz, δ ppm (*J* = Hz)].

	1	2	3	4	5	6
1	3.56 ddd (9.6, 7.1, 7.1)	3.60 m	3.47 ddd (9.5, 7.8, 7.2)	3.01 dd (9.7, 7.2, 7.2)	3.01 ddd (9.4, 7.1, 7.1)	2.96 ddd (9.3, 7.3, 7.3)
2a	2.45 ddd (14.7, 7.1, 5.3)	2.49 ddd (14.6, 7.0, 5.5)	2.40 m	2.10 ddd (13.1, 7.2, 7.0)	2.09 ddd (13.4, 7.1, 7.1)	2.10 ddd (13.8, 7.3, 7.1)
2b	1.51 ddd (14.7, 10.0, 7.1)	1.54 dd (14.6, 10.2, 7.0)	1.60 ddd (14.0, 10.1, 7.8)	1.75 ddd (13.1, 9.3, 7.2)	1.74 ddd (13.4, 9.3, 7.1)	1.71 ddd (13.8, 9.4, 7.3)
3	4.07 m	4.10 m	4.07 m	4.51 t (9.3, 7.0)	4.51 dd (9.3, 7.1)	4.48 dd (9.4, 7.1)
5	2.26 t (9.6)	2.28 dd (9.8, 9.2)	2.28 dd (10.2, 9.7)	2.90 t (10.1, 9.7)	2.91 dd (10.2, 9.4)	2.84 m
6	4.86 dd (9.6, 9.2)	4.91 dd (9.8, 9.2)	4.80 m	4.34 t (10.1)	4.35 t (10.2)	4.16 dd (10.3, 9.2)
7	3.15 br t (9.2)	3.17 m	3.20 m	3.26 m	3.28 m	2.87 m
8	5.12 m	5.12 m	5.12 m	5.12 m	5.15 m	3.87 ddd (9.3, 4.8, 4.3)
9a	2.67dd (15.0, 4.5)	2.71 dd (15.0, 4.8)	2.75 dd (14.5, 5.0)	2.73 dd (14.5, 4.9)	2.73 dd (14.5, 5.0)	2.67 dd (13.8, 4.8)
9b	2.38 br d (15.0)	2.42 br d (15.0)	2.38 dd (14.5, 3.2)	2.38 br d (14.5)	2.40 dd (14.5, 2.9)	2.25 dd (13.8, 4.3)
13a	6.07 d (2.8)	6.10 d (3.3)	6.11 d (3.0)	6.18 d (2.5)	6.13 d (3.1)	6.16 d (3.2)
13b	5.59 d (2.8)	5.57 d (3.3)	5.45 d (3.0)	5.59 d (2.5)	5.65 d (3.1)	6.14 d (3.2)
14a	5.08 br s	5.10 br s	5.12 br s	5.15 br s	5.17 br s	5.09 br s
14b	4.75 br s	4.77 br s	4.85 br s	4.91 br s	4.92 br s	4.97 br s
15a	4.14 d (11.7)	4.19 d (11.7)	4.02 d (11.8)	5.44 br s	5.44 br s	5.40 br s
15b	3.80 d (11.7)	3.83 d (11.7)	3.81 d (11.8)	5.34 br s	5.34 br s	5.31 br s
3′a	6.28 br s	6.18 br s	6.31 br s	6.13 d (2.5)	6.31 br s	-
3′b	5.94 br s	5.73 br s	5.97 br s	5.73 d (2.5)	5.97 br s	-
4′	4.28 s (2H)	1.98 s	4.31 s (2H)	1.97 s	4.31s (2H)	-

**Table 2:** ^13^C-NMR data of compounds **1–3**, **5** and **6** (MeOH-*d_4_*, 125 MHz, δ ppm).

Position	1	2	3	5	6
1	48.73	48.95	46.90	46.18	45.93
2	39.80	40.02	39.15	40.01	40.01
3	76.94	76.99	77.81	74.14	74.11
4	85.77	85.84	85.82	154.04	154.26
5	59.39	59.64	57.53	52.02	51.81
6	78.45	78.52	78.84	80.30	80.87
7	47.43	47.60	48.17	48.45	51.66
8	75.47	75.57	75.63	75.63	73.07
9	35.74	35.80	37.48	37.66	42.84
10	144.71	145.05	144.68	144.04	144.63
11	139.11	139.52	139.23	139.71	140.53
12	170.97	170.84	171.11	171.21	172.0
13	122.53	122.01	122.45	122.39	122.95
14	117.71	117.48	117.27	118.15	117.03
15	50.04	50.08	64.35	112.72	112.15
1′	166.65	167.86	166.55	166.55	-
2′	141.61	137.76	141.91	141.92	-
3′	126.25	126.92	125.97	125.97	-
4′	61.53	18.31	61.62	61.63	-

**Table 3:** Antiproliferative activity of the isolated sesquiterpenoids, flavonoids, and coumarin.

		Inhibition of Cell Proliferation (%) ± SEM [Calculated IC_50_ (μM)]		
Compound	Conc.	SiHa	HeLa	MDA-MB-231
Chlorojanerin (1)	10 μM	82.10 ± 0.97	51.83 ± 2.43	90.55 ± 1.27
	30 μM	92.93 ± 0.35	77.08 ± 0.80	92.00 ± 0.68
		[6.71]	[11.37]	[2.21]
19-Deoxychlorojanerin (2)	10 μM	90.93 ± 1.32	96.17 ± 0.38	96.14 ± 0.51
	30 μM	95.80 ± 0.15	96.14 ± 0.48	97.42 ± 0.36
		[5.54]	[4.86]	[2.88]
15-Hydroxyjanerin (3)	10 μM	<10	<10	<10
	30 μM	<10	<10	<10
Aguerin B (4)	10 μM	89.09 ± 1.54	28.87 ± 0.83	96.23 ± 0.45
	30 μM	97.92 ± 0.41	97.50 ± 0.31	96.64 ± 0.71
		[5.70]	[12.64]	[4.26]
Cynaropicrin (5)	10 μM	63.03 ± 1.25	34.36 ± 2.37	92.16 ± 0.68
	30 μM	90.75 ± 0.55	68.26 ± 1.85	93.45 ± 0.27
		[8.39]	[12.99]	[4.80]
Eleganin (6)	10 μM	64.97 ± 2.06	16.54 ± 1.48	89.44 ± 1.36
	30 μM	90.89 ± 0.43	73.99 ± 1.68	92.09 ± 0.9
		[7.57]	[19.62]	[6.86]
Apigenin	10 μM	<10*	18.81 ± 0.80	<10
	30 μM	29.92 ± 1.75	70.43 ± 1.09	28.42 ± 1.04
Jaceosidine	10 μM	18.88 ± 2.99	<10	<10
	30 μM	60.08 ± 0.74	75.18 ± 0.68	40.04 ± 1.68
6-Methoxyluteolin	10 μM	14.16 ± 1.49	88.00 ± 0.63	<10
	30 μM	35.20 ± 2.08	93.74 ± 0.65	26.55 ± 2.12
			[4.50]	
Scopoletin	10 μM	<10	<10	12.09 ± 2.59
	30 μM	17.18 ± 2.18	<10	15.85 ± 1.85
Cisplatin	10 μM	88.64 ± 0.50	42.61 ± 2.33	67.51 ± 1.01
	30 μM	90.18 ± 1.78	99.93 ± 0.26	87.75 ± 1.10
		[7.84]	[12.43]	[3.74]

* Antiproliferative activities less than 10% are considered negligible and not given numerically.

## Supplementary Materials

The following are available online at https://www.mdpi.com/1420-3049/24/17/3165/s1, ^1^H-NMR spectra of compounds **1**–**6**, and apigenin, 6-methoxyluteolin, jaceosidine and scopoletin are available online.
