# Supplementary material for: Sesquiterpene Lactones and Flavonoids from Psephellus pyrrhoblepharus with Antiproliferative Activity on Human Gynecological Cancer Cell Lines
Source: Molecules. 2019 Aug 30;24(17):3165. doi: 10.3390/molecules24173165 (PMC6749316; doi:10.3390/molecules24173165)
Supplement: Supplementary file 1 [file molecules-24-03165-s001.pdf]

SUPPORTING INFORMATION

Sesquiterpene lactones and flavonoids from *Psephellus pyrrhoblepharus* with antiproliferative activity on human gynecological cancer cell lines

Pelin Tastan [1], Zsuzsanna Hajdú [2], Norbert Kúsz [2], István Zupkó [3,4], Izabella Sinka [3], Bijen Kivcak [1] and Judit Hohmann [2,4*]

[1] Department of Pharmacognosy, Faculty of Pharmacy, Ege University, İzmir, Turkey
[2] Institute of Pharmacognosy, Interdisciplinary Excellence Centre, University of Szeged, Szeged, Hungary
[3] Institute of Pharmacodynamics and Biopharmacy, Interdisciplinary Excellence Centre, University of Szeged, Szeged, Hungary
[4] Interdisciplinary Centre of Natural Products, University of Szeged, Szeged, Hungary
* Correspondence: hohmann@pharm.u-szeged.hu; Tel.: +36-62-545 558

Content

Figure S1. ^{1}H NMR spectrum of chlorojanerin (**1**)..3

Figure S2. ^{1}H NMR spectrum of 19-deoxychlorojanerin (**2**)..3

Figure S3. ^{1}H NMR spectrum of 15-hydroxyjanerin (**3**)..4

Figure S4. ^{1}H NMR spectrum of aguerin B (**4**)..4

Figure S5. ^{1}H NMR spectrum of cynaropicrin (**5**)..5

Figure S6. ^{1}H NMR spectrum of eleganin (**6**)..5

Figure S7. ^{1}H NMR spectrum of apigenin..6

Figure S8. ^{1}H NMR spectrum of 6-methoxyluteolin..6

Figure S9. ^{1}H NMR spectrum of jaceosidine..7

Figure S10. ^{1}H NMR spectrum of scopoletin..7

Figure S11. Separation of sesquiterpenoids and flavonoids by Sephadex LH-20 gel filtration of fraction C_5..8

Figure S12. Separation by gel filtration of fraction D, subfraction II on Sephadex LH-20..8

Figure S13. RPC fractionation of fraction D, subfraction II, gel filtration 3+4..9

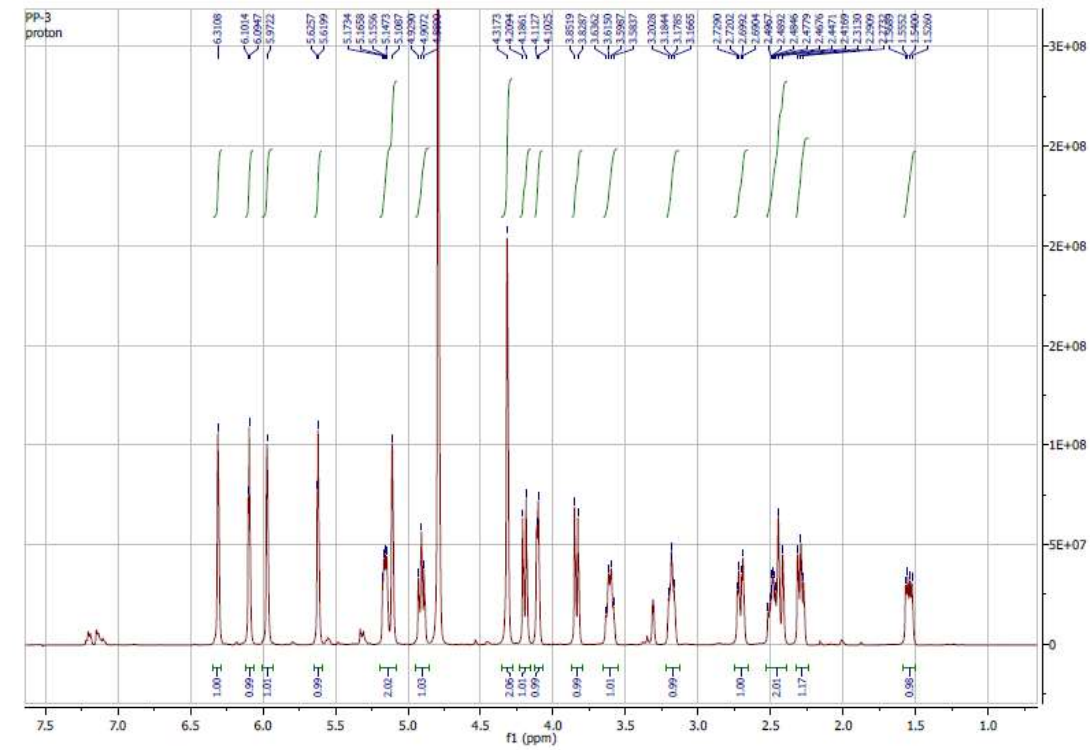

Figure S1. ^{1}H NMR spectrum of chlorojanerin (**1**) (500 MHz, MeOH-d_4)

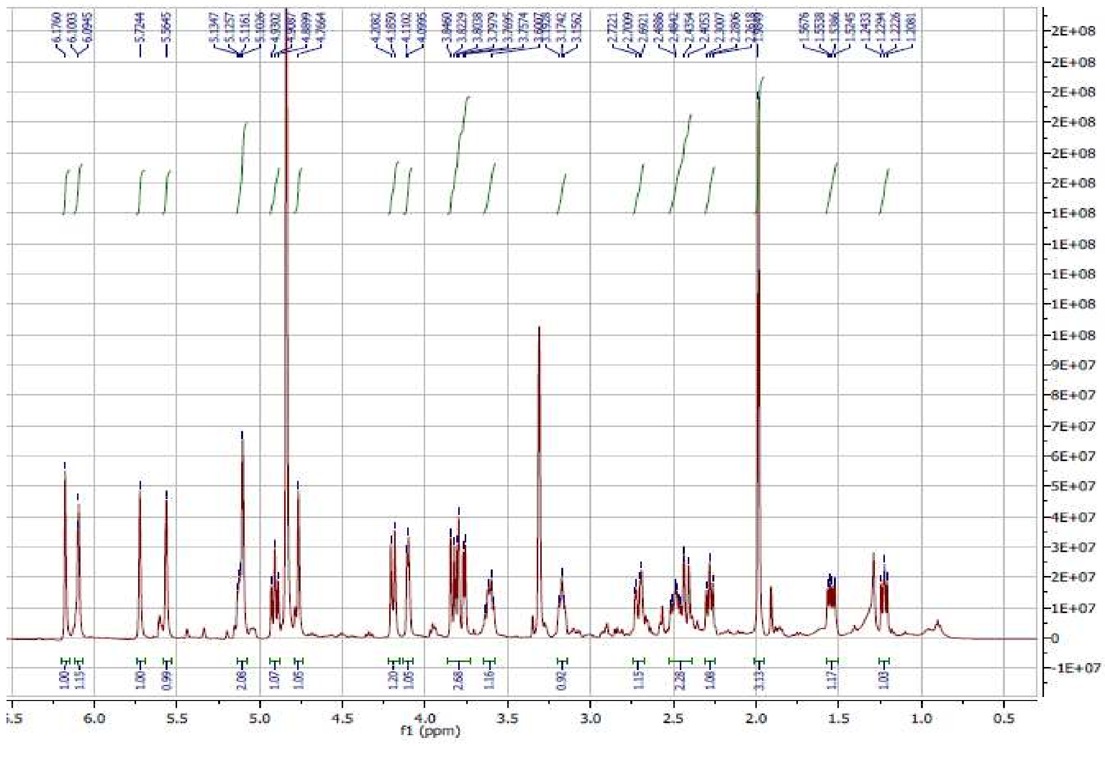

Figure S2. ^{1}H NMR spectrum of 19-deoxychlorojanerin (**2**) (500 MHz, MeOH-d_4)

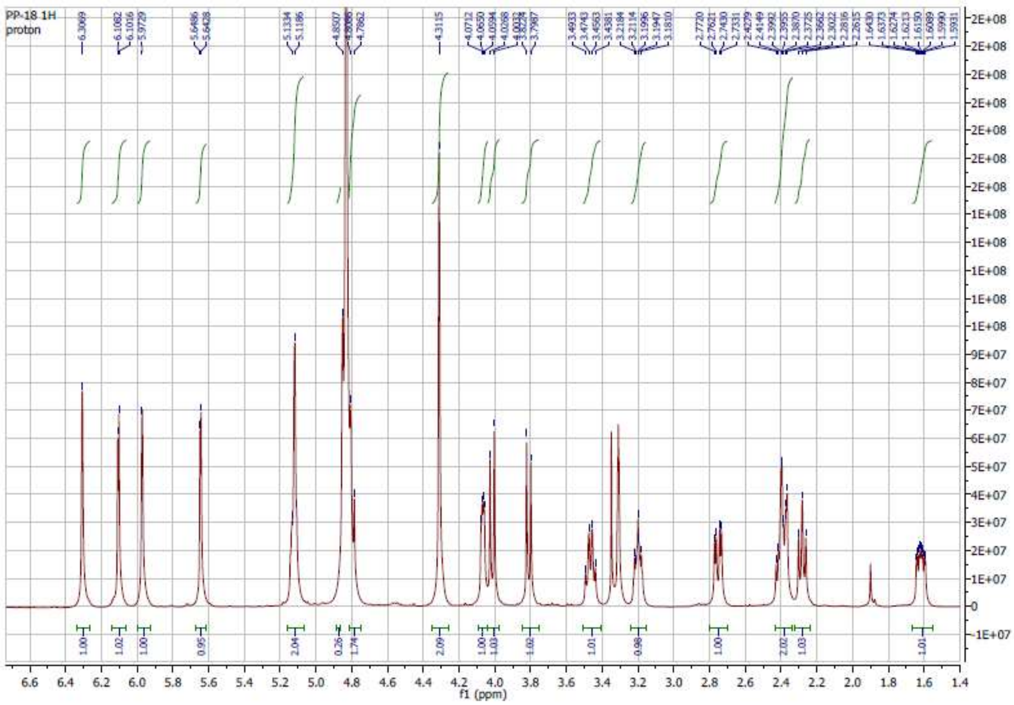

Figure S3. ^{1}H NMR spectrum of 15-hydroxyjanerin (**3**) (500 MHz, MeOH-d_4)

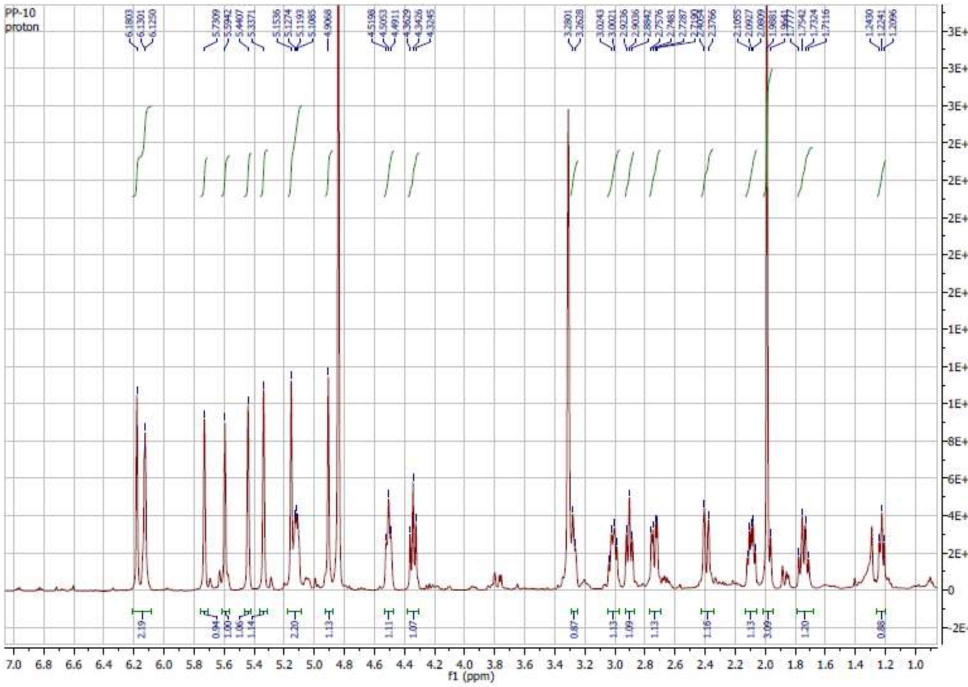

Figure S4. ^{1}H NMR spectrum of aguerin B (**4**) (500 MHz, MeOH-d_4)

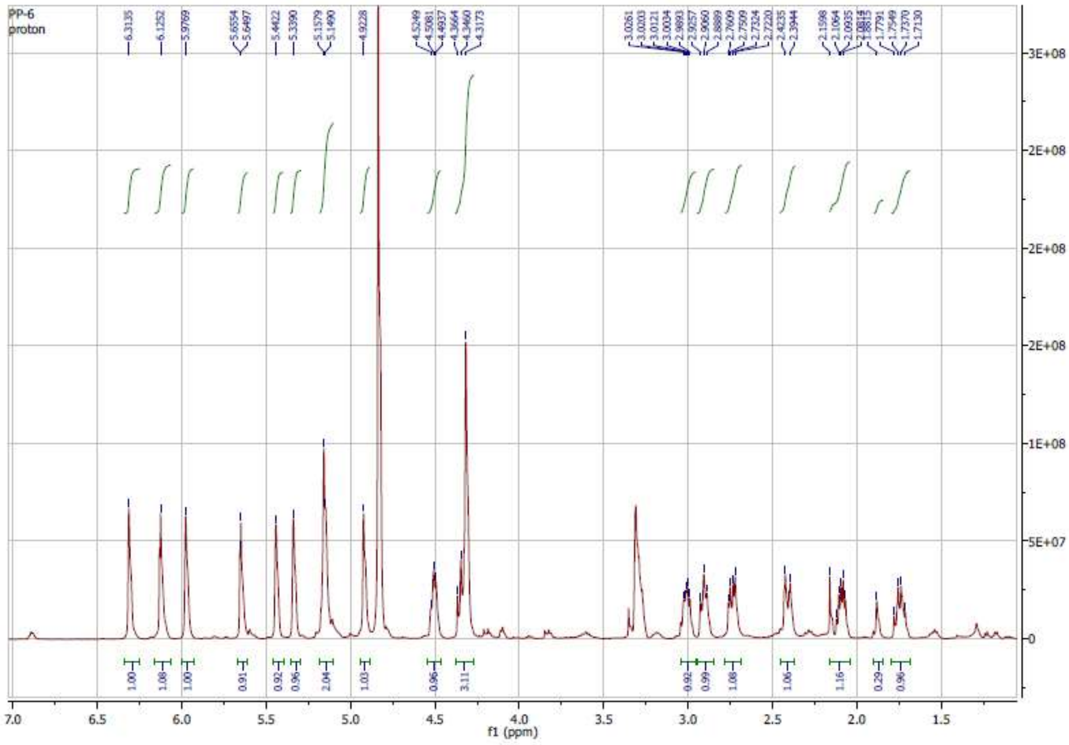

Figure S5. ^{1}H NMR spectrum of cynaropicrin (**5**) (500 MHz, MeOH-d_4)

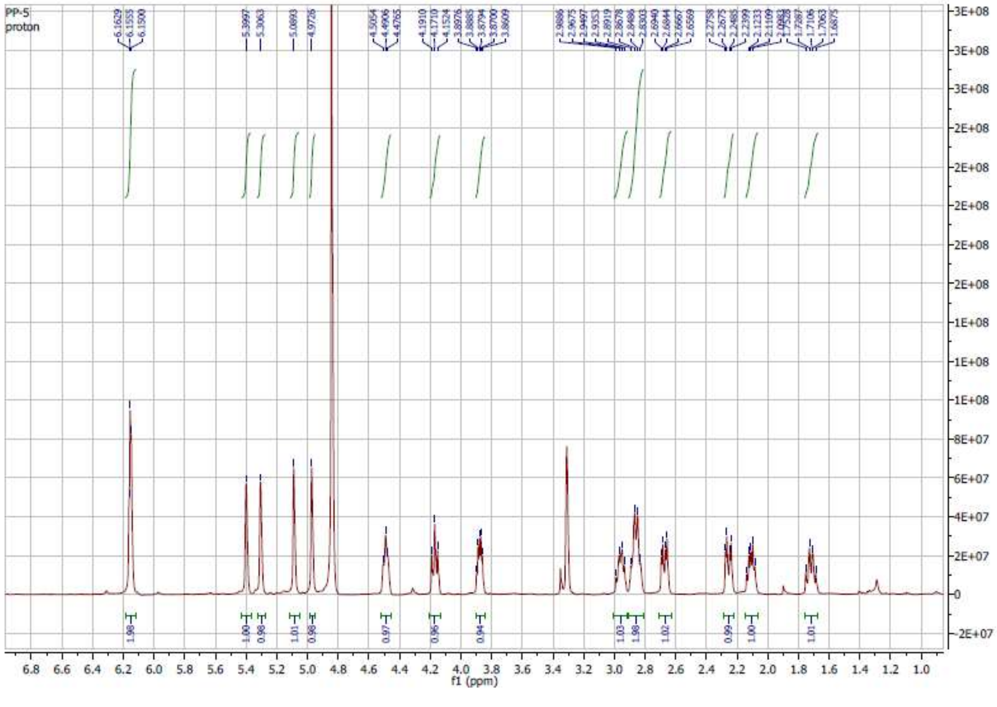

Figure S6. ^{1}H NMR spectrum of eleganin (**6**) (500 MHz, MeOH-d_4)

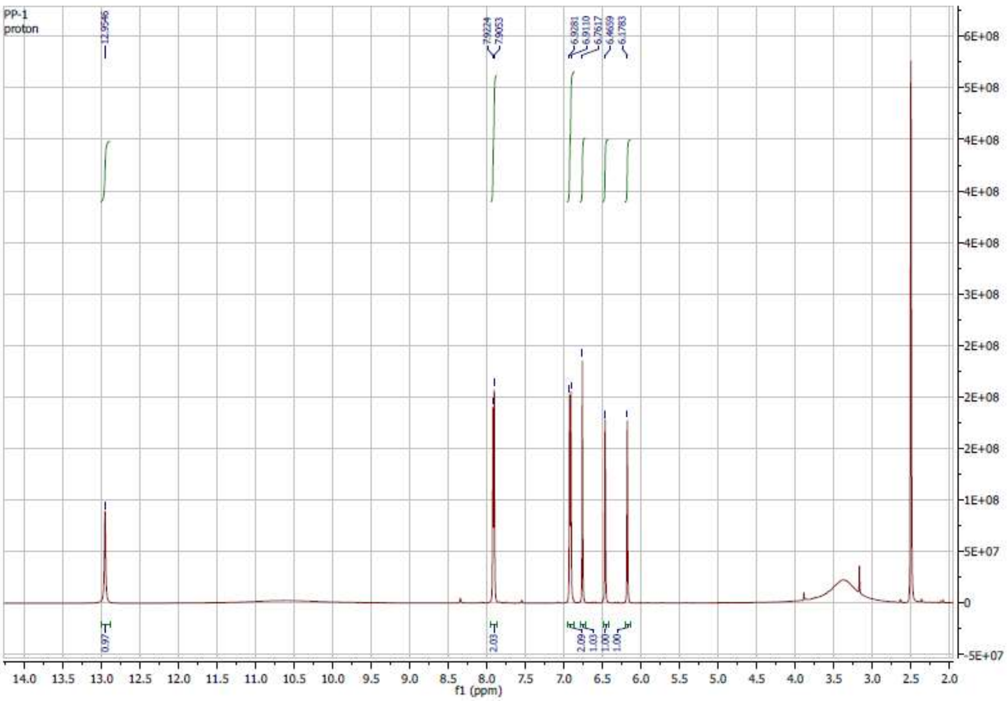

Figure S7. ^{1}H NMR spectrum of apigenin (500 MHz, MeOH-*d*$_4$)

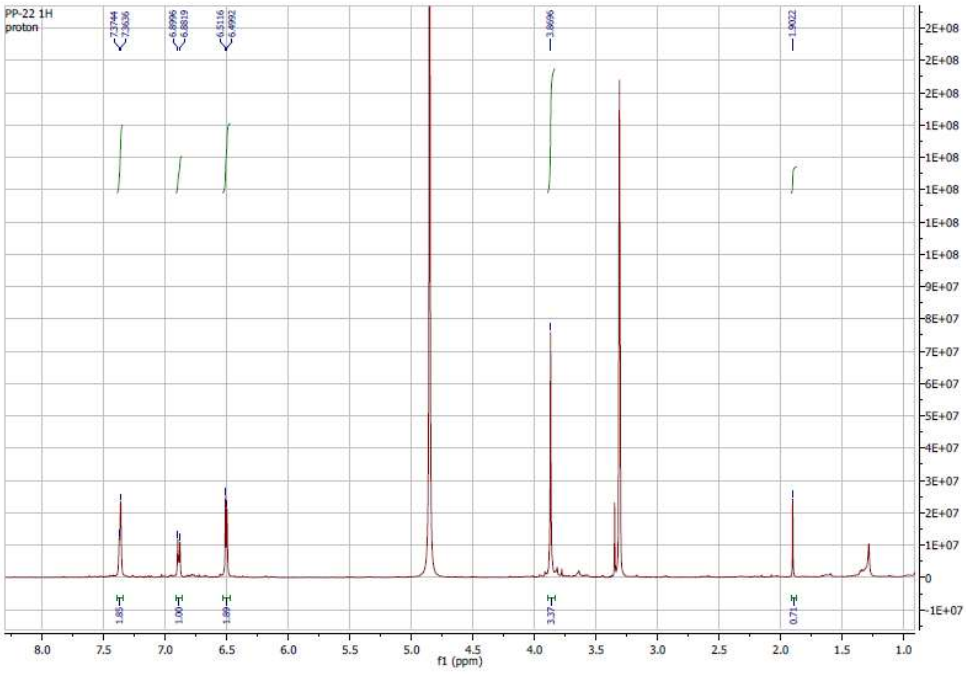

Figure S8. ^{1}H NMR spectrum of 6-methoxyluteolin (500 MHz, MeOH-*d*$_4$)

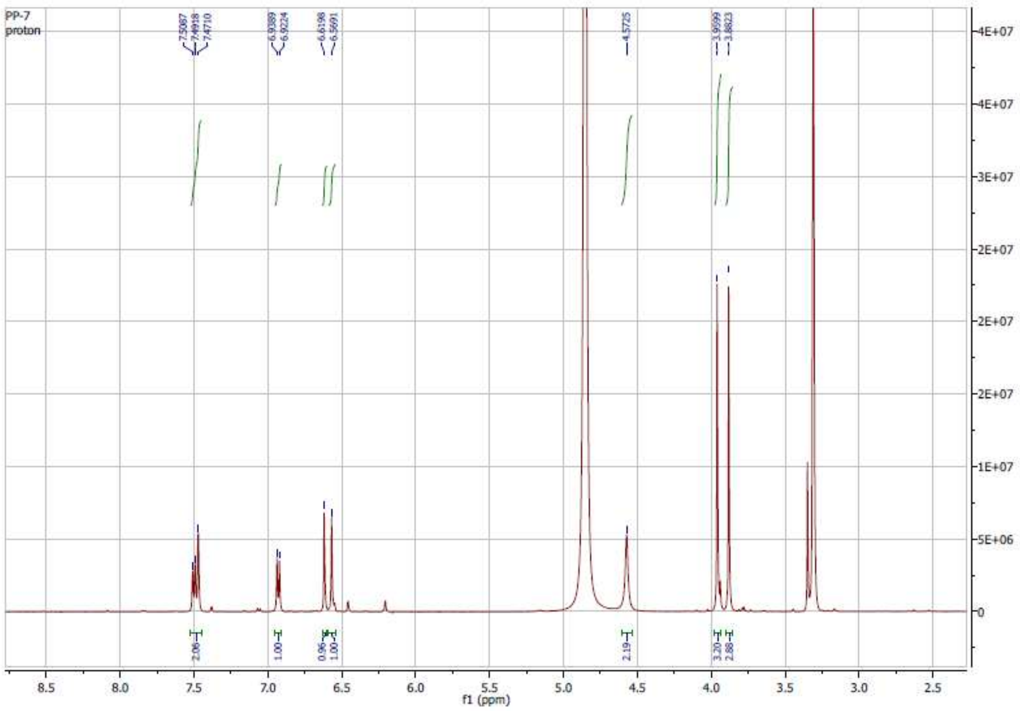

Figure S9. ^{1}H NMR spectrum of jaceosidine (500 MHz, DMSO-d_6)

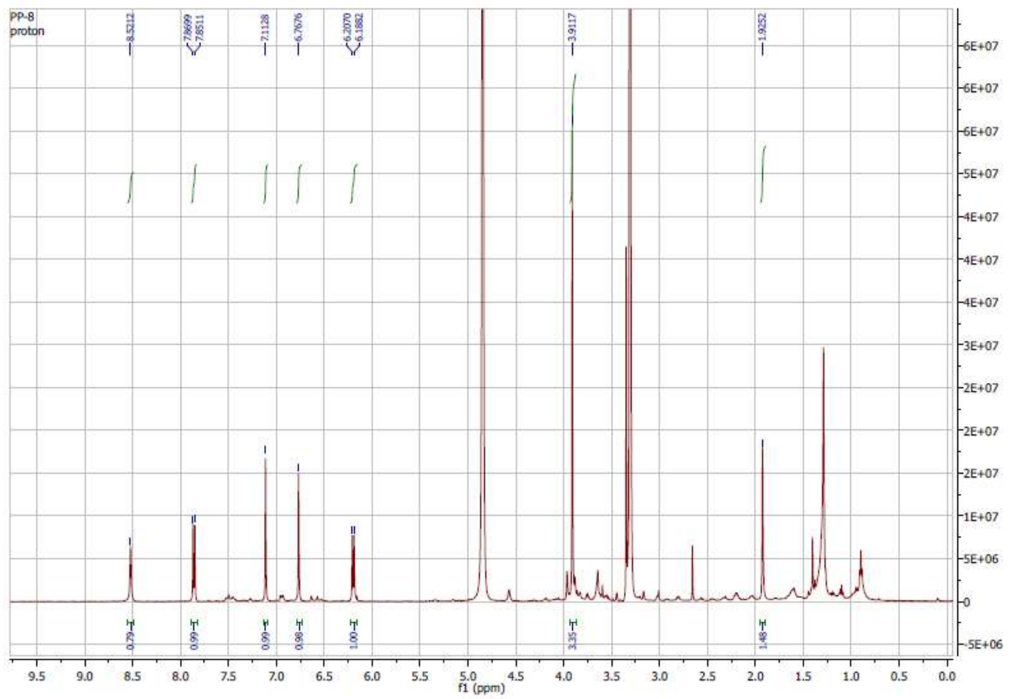

Figure S10. ^{1}H NMR spectrum of scopoletin (500 MHz, MeOH-d_4)

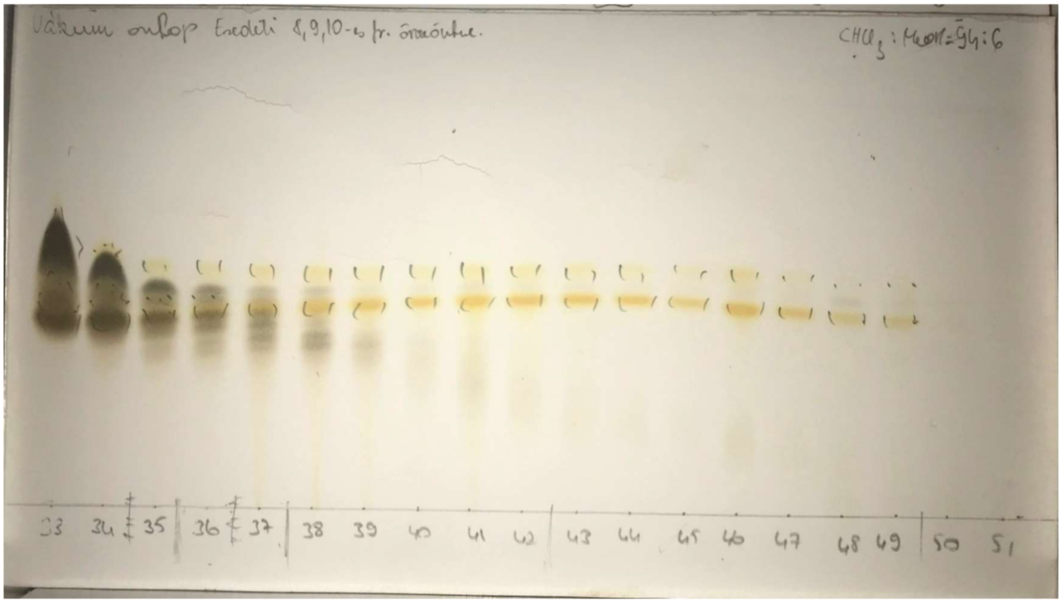

Figure S11. Separation of sesquiterpenoids (start points 1-5) and flavonoids (start points 6-17) by Sephadex LH-20 gel filtration of fraction C_5.
(Silica gel F_{254}, developing system $CHCl_3$:MeOH 94:6)

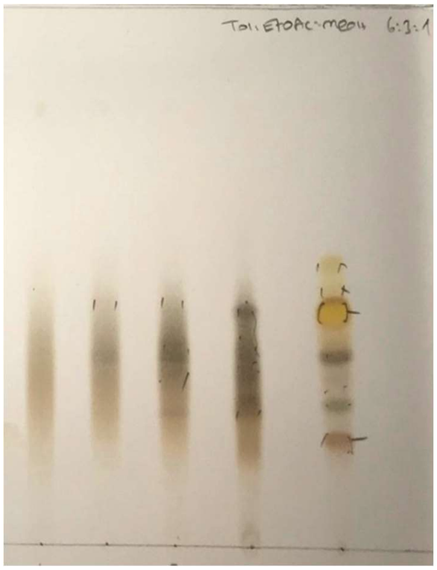

1 2 3 4 5

Figure S12. Separation by gel filtration of fraction D, subfraction II on Sephadex LH-20.
(Silica gel F_{254}, developing system toluene:EtOAc:MeOH 6:3:1)

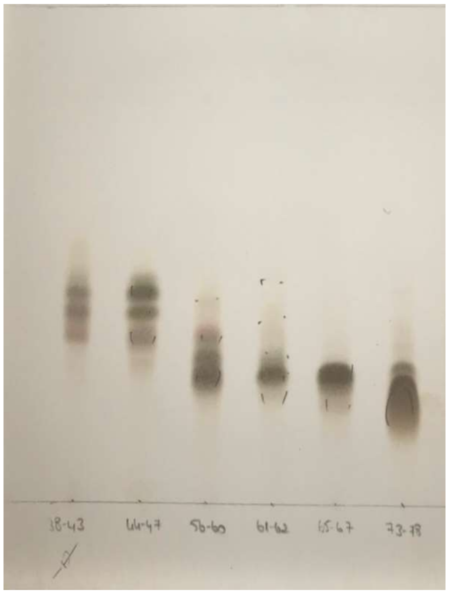

Figure S13. RPC fractionation of fraction D, subfraction II, gel filtration 3+4.
(Silica gel F_{254}, developing system toluene:EtOAc:MeOH 6:3:1).
